# The Role of Inflammation in the Pathophysiology of Heart Failure

**DOI:** 10.3390/cells14141117

**Published:** 2025-07-21

**Authors:** Marwan Amara, Ohad Stoler, Edo Y. Birati

**Affiliations:** 1 The Kittner-Davidai Division of Cardiovascular Medicine, Tzafon Medical Center, Tiberias 1520801, Israel; maamara@tzmc.gov.il; 2 Azrieli Faculty of Medicine, Bar Ilan University, Ramat Gan 5290002, Israel; ostoler@tzmc.gov.il

**Keywords:** inflammation, HFpEF, HErEF, heart failure, epidemiology, pathophysiology

## Abstract

Heart failure (HF), a prevalent global health issue characterized by the heart’s impaired ability to pump or fill blood, affects millions worldwide and continues to pose significant challenges despite advancements in treatment. This review delves into the critical and increasingly recognized role of inflammation in the development and progression of this complex syndrome. While the incidence of HF has seen a decline in some regions due to improved cardiac care, its overall prevalence is rising, particularly among younger adults and those with heart failure with a preserved ejection fraction (HFpEF). Given the persistently high rates of hospitalization and mortality associated with HF, understanding the underlying mechanisms, including the contribution of inflammation, is crucial for identifying novel therapeutic strategies. Inflammation in heart failure is a multifaceted process involving the activation of the immune system, both innate and adaptive, and encompasses various mechanisms such as the release of pro-inflammatory mediators, endothelial dysfunction, and neurohormonal activation. Myocardial damage triggers the innate immune response, while humoral immunity and chronic systemic inflammation, often linked to cardiovascular risk factors and autoimmune diseases, also play significant roles. Notably, heart failure and inflammation have a reciprocal relationship, with HF itself contributing to inflammatory processes within the cardiac tissue and systemically. Understanding these intricate pathways, including the involvement of specific immune cells and molecular mediators, is essential for comprehending the pathogenesis of heart failure and exploring potential therapeutic interventions. The review further examines various inflammatory biomarkers that have been implicated in heart failure, such as cytokines (including TNF-α and IL-1) and C-reactive protein (CRP). While these markers often correlate with the severity and prognosis of HF, clinical trials targeting specific inflammatory mediators have largely yielded disappointing results, highlighting the complexity of the inflammatory response in this context. The exploration of these biomarkers and the challenges encountered in translating anti-inflammatory strategies into effective treatments underscore the need for continued research to unravel the precise role of inflammation across different HF subtypes and to develop more targeted and effective anti-inflammatory therapies.

## 1. Introduction

Heart failure (HF) is a clinical syndrome characterized by the reduced ability of the heart to pump and/or fill with blood, which may result in an inadequate cardiac output or in preserved cardiac output secondary to compensatory neurohormonal activation and increased left ventricular filling pressure [1].

Heart failure is commonly classified by the left ventricular ejection fraction (EF) into three groups: (1) heart failure with a reduced ejection fraction (HFrEF; EF < 40%), (2) heart failure with a mildly reduced ejection fraction (HFmrEF; EF = 41–49%), and (3) heart failure with a preserved ejection fraction (HFpEF; EF > 50%) [2]. Additional classifications include a staging system aimed at identifying patients at risk of developing HF—Stages A and B (pre-symptomatic)—as well as those with established clinical symptoms—Stages C and D. The New York Heart Association (NYHA) functional classification further categorizes HF severity based on the level of physical activity that induces symptoms (Table 1) [3].

HF represents a significant global health issue affecting approximately 64 million people worldwide. It is estimated that the entity impacts about 2% of the population and over 10% of the elderly population in the United States, with 550,000 new cases annually [2], and higher incidence and mortality rates among African Americans and other ethnic minorities [4].

While the incidence of HFrEF has declined in high-income countries—primarily due to timely and effective cardiac catheterizations in the setting of acute myocardial injury [1,5]—the overall prevalence and incidence of HF have continued to rise. This increase is driven by an aging population, improved survival from other cardiovascular diseases, and the growing prevalence of cardiovascular risk factors such as hypertension, diabetes, and obesity [4]. The trend is particularly evident in a growing proportion of cases with preserved ejection fraction (HFpEF) [4]. Despite therapeutic advancements, HF continues to impose a substantial burden in terms of hospitalizations and mortality [4].

Over the last two decades, countless experiments and clinical data have shown the pivotal role of inflammation in the development and progression of cardiovascular disease [6,7,8,9]. Most of these theories and clinical trials focus on the relationship between inflammation and atherosclerosis of the coronary arteries or the development of ischemic cardiomyopathy. Few trials targeted major inflammatory mediators in order to explore better therapeutic targets for the prevention and treatment of HF [10,11]. In this review, we will discuss the role of inflammation in the pathogenesis of heart failure [12].

## 2. Pathophysiology

Inflammation is part of the physiological response to various stimuli, such as harmful pathogens and damaged cells. It primarily involves immune cells and molecular mediators, aiming to eliminate the cause of cell injury, clear damaged cells, and initiate tissue repair [13].

Inflammation has been recognized as a common pathophysiological feature of both acute and chronic HF [14,15]. However, its contribution appears to vary depending on the type of HF [16]. In acute HF, inflammation is mainly attributed to apoptotic and necrotic damage and subsequent tissue repair, whereas in chronic HF it is associated with fibrosis and remodeling of the extracellular matrix (ECM) [16]. Moreover, HF has been associated with worsening disease advancement and development of adverse outcomes and was found to be a prognostic factor, regardless of conventional measures like left ventricular ejection fraction (LVEF) or the New York Heart Association (NYHA) functional classification [17,18].

Many clinical trials, such as RELAX, BIOSTAT-CHF, TIME-CHF, and ASCEND-HF, have shown a correlation between chronic inflammation and the initiation and progression of HF. However, studies on anti-inflammatory therapies have failed to show clinical benefit in this patient population [14,15,19,20].

Various mechanisms contribute to the pathogenesis of heart failure:

### 2.1. Immune System Activation

Inflammation in HF can be classified by its type (sterile, para-inflammation, or chronic inflammation) or according to the sources of inflammation (local vs. systemic). Inflammation can also be classified by its duration (acute, subacute, chronic), associated risk factors (diabetes, obesity, hypertension, hyperlipidemia, smoking, and physical inactivity), and etiologies (autoimmune disease, infections, aging, and tissue injury) (see Table 2) [21].

A typical inflammatory response consists of four components:

(1) Inflammatory inducers, classified as exogenous [microbial inducers, including pathogen-associated molecular patterns (PAMPs), and non-microbial inducers: allergens, toxic compounds, and irritants] or endogenous inducers [including danger-associated molecular patterns (DAMPs) such as cells, tissues, plasma, and extracellular matrix-derived products].
(2) Sensors that detect inflammatory inducers, such as pattern recognition receptors (PRRs) or Toll-like receptor 4 (TLR4), and the nucleotide-binding oligomerization domain-like receptor with a pyrin domain 3 (NLRP3) inflammasome, play a crucial role in initiating the inflammatory response.
(3) Inflammatory mediators induced by the sensors (vasoactive amines and peptides, fragments of complement components, lipid mediators, proteolytic enzyme chemokines, and cytokines).
(4) The target tissues that are impacted by the inflammatory mediators [22,23].

#### 2.1.1. The Innate Immune System

**Figure 1:**
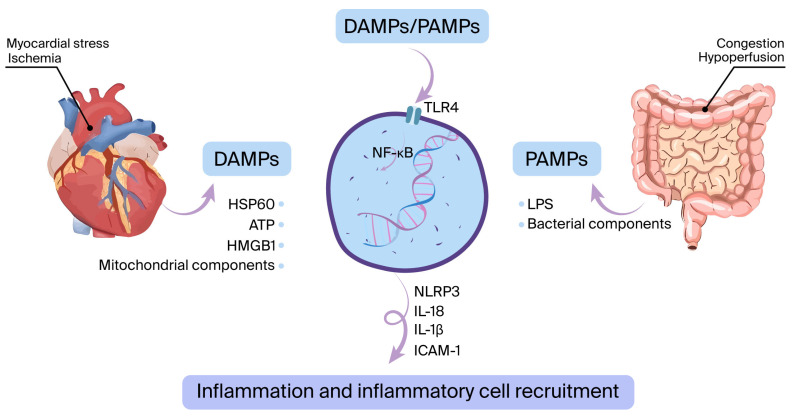
The innate immune system is activated when damage-associated molecular patterns (DAMPs) or pathogen-associated molecular patterns (PAMPs) interact with pattern recognition receptors such as toll-like receptor 4 (TLR4). During ischemia or other forms of myocardial stress, DAMPs are released. Additionally, bacterial components like lipopolysaccharide (LPS) can cross from the gut into the bloodstream, functioning as PAMPs. This interaction initiates a cascade leading to the upregulation of inflammatory mediators—such as interleukin-6 (IL-6), IL-1β, and ICAM-1—via the NF-κB signaling pathway and activation of the NLRP3 inflammasome.

The innate immune system is activated mostly due to myocardial ischemia (acute coronary syndrome) or other types of myocardial damage (such as myocarditis, acute decompensated heart failure, infiltrative disease, etc.) [24]. This activation triggers the emergence of DAMPs, which consist of elements like heat-shock protein-60 (HSP60), high mobility group box 1 (HMGB1), and mitochondrial components. In addition, PAMPs such as bacterial products and lipopolysaccharides (LPS) may pass into circulation via the digestive system and stimulate pattern recognition receptors (PRRs), primarily TLR4 [25,26]. This process leads to the stimulation of nuclear factor kappa B (NFκB) and the NLRP3 inflammasome, triggering the production of proinflammatory cytokines. These include interleukin (IL)-6, IL-1β, tumor necrosis factor-alpha (TNF-α), intercellular adhesion molecule-1 (ICAM-1), adenosine triphosphate (ATP), HMGB1, and HSP60 (Figure 1) [27,28,29].

#### 2.1.2. Humoral Immunity

Humoral immunity has a significant role in the progression of heart failure. Several studies have shown the presence of anti-cardiac autoantibodies in up to 60% of patients with non-ischemic cardiomyopathy and in 90% of patients on durable left ventricular assist device (LVAD) support [30]. Some studies suggest that these antibodies may act directly against cardiac proteins, including β1-adrenergic receptors, mitochondrial components, troponin I, myosin, and the sarcolemma Na-K-ATPase enzyme [31,32].

Moreover, animal studies have shown a correlation between free light chain (kappa and lambda) levels in chronic inflammatory conditions and myocardial apoptosis and fibroblasts [33,34]. Among patients with recent hospitalization with HF, elevated levels of light chains were associated with a high risk of mortality [35].

#### 2.1.3. Chronic Inflammation

Cardiovascular risk factors, including obesity, chronic inflammatory disease, diabetes, metabolic syndrome, and aging, are associated with chronic inflammation [36,37]. Chronic inflammation is caused by the persistence of inflammatory triggers, disabling the resolution of the inflammatory phase by a vicious cycle of inflammation and the primary pathological condition [38]. The inflammatory reaction triggers myofibroblast activation that can further induce a subsequent inflammatory response. This response includes the release of chemokines that facilitate the transmigration of a variety of immune cells as a result of NLRP3 inflammasome activity and IL-1ß release [39].

#### 2.1.4. Systemic Inflammation Secondary to Chronic Inflammatory Diseases

Chronic inflammatory diseases such as rheumatoid arthritis (RA) and systemic lupus erythematosus (SLE) are associated with chronic systemic inflammation [40]. Many studies have demonstrated the impact of these inflammatory diseases on the occurrence and progression of HF [40].

Several mechanisms have been implicated in the development of HF in these patients, including the occurrence of HF in these patients, including the following:

(1) Atherosclerosis in early stages: For example, patients with RA are more likely to experience rapid progression of atherosclerosis within the first six years following diagnosis, potentially leading to ischemic cardiomyopathy [41,42].
(2) Autonomic nervous system: Several inflammatory cytokines target the autonomic center in the brain and increase sympathetic activity. Furthermore, the circulatory pro-inflammatory cytokines also stimulate the β2 adrenal receptors in circulating lymphocytes and monocytes. Although the “inflammatory reflex” may reduce cytokine activity through a negative feedback loop, it can also result in enhanced sympathetic activation, which in turn may amplify the inflammatory response. Moreover, the increase in sympathetic activity results in parasympathetic–sympathetic imbalance, resulting in an increased risk of arrhythmia [43].

#### 2.1.5. The Impact of Heart Failure on Further Inflammatory Response

Heart failure and inflammation have a reciprocal relationship, forming a vicious cycle in which each condition exacerbates the other. Heart failure (both with preserved and reduced ejection fraction) may impair oxygen delivery to peripheral organs. In addition, in response to mechanical stress and cell death, cardiomyocytes release sterile inflammation-related cytokines (“cardio-cytokines”). This inflammatory and hemodynamic cascade may affect multiple organs, including the spleen, bone marrow, adipose tissue, and skeletal muscle, either directly or indirectly. This organ dysfunction may further aggravate the inflammatory cycle, leading to increased cell death, myocardial fibrosis, and structural remodeling of the heart [44].

### 2.2. Endothelial Inflammation

**Figure 2:**
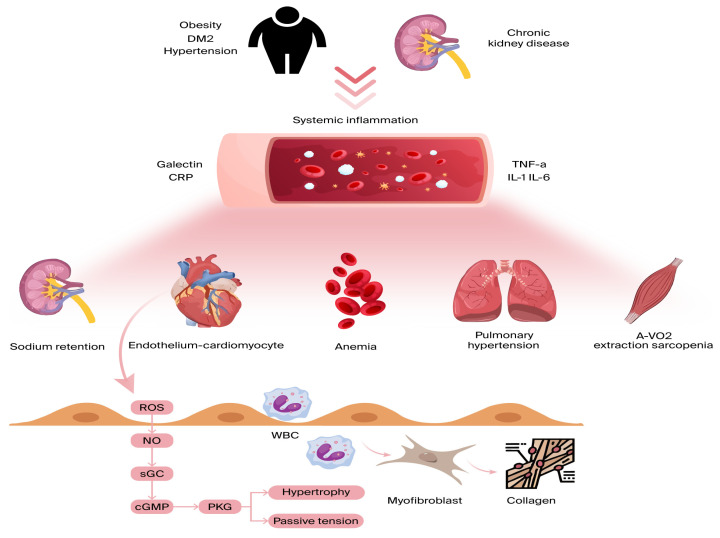
Comorbidities lead to ongoing low-grade inflammation, affecting various organs beyond the heart. Inflammatory cytokines hinder skeletal muscle oxygen extraction during exercise, exacerbate anemia and sarcopenia, promote sodium retention in the kidneys, and raise pulmonary pressures during exercise due to pulmonary vasoconstriction. All these factors contribute to dyspnea and reduced exercise tolerance in heart failure. On a myocardial level, microvascular endothelial inflammation boosts the expression of adhesion molecules, drawing in circulating leukocytes and leading to myofibroblast formation and interstitial collagen buildup. Endothelial inflammation also triggers reactive oxygen species (ROS) production and reduced nitric oxide (NO) bioavailability. This sequence lowers soluble guanylate cyclase (sGC) activity, cyclic guanosine monophosphate (cGMP) levels, and protein kinase G (PKG) activity, resulting in cardiomyocyte stiffness and hypertrophy. A-VO2: arteriovenous oxygen difference; CRP: C-reactive protein; DM2: diabetes mellitus; and TNF a: tumor necrosis factor alpha.

Inflammation affecting the coronary microvascular endothelium results in a reduction in nitric oxide (NO) availability and a surge in reactive oxygen species (ROS) [45]. As NO levels decrease, protein kinase G (PKG) activity is suppressed, leading to several adverse effects. One key consequence is the hyperphosphorylation of Titin, a large cytoskeletal protein responsible for acting as a biomechanical regulator within cardiomyocytes. This alteration heightens resting tension and cellular stiffness, negatively impacting myocardial function. Additionally, the reduction in PKG activity plays a role in cardiomyocyte hypertrophy, further worsening diastolic dysfunction [45,46] (Figure 2).

Beyond these effects, endothelial inflammation triggers additional pathological changes. It enhances the production of cell-binding molecules, such as VCAM-1 and E-selectin, which activate circulating monocytes and induce interstitial collagen accumulation [47]. Moreover, inflammation can disrupt microvascular vasodilation, stimulate fibroblast and myofibroblast activity [45], and lead to capillary rarefaction, causing further complications [48].

### 2.3. Neurohormonal Activation

Chronic inflammation activates neurohormonal systems like the renin–angiotensin–aldosterone system (RAAS). The pro-inflammatory and profibrotic properties of angiotensin II, a key component of the RAAS, have been implicated in the pathogenesis of HF [49]. Activation of the RAAS system contributes to immune cell infiltration, further exacerbating inflammation and fibrosis responses in the heart [50]. Specifically, macrophage mineralocorticoid receptors (MRs) play a crucial role in shifting macrophage polarization from the anti-inflammatory M2 phenotype toward the pro-inflammatory M1 phenotype, thereby sustaining inflammatory responses and promoting tissue remodeling. It has been shown that blocking or deleting macrophage MR protects against cardiovascular remodeling, even when aldosterone levels are elevated [51].

Oxidative stress may also amplify inflammation by directly activating MRs or by lowering the threshold for glucocorticoid activation of macrophages [52,53]. Additionally, some studies have found that aldosterone excess reduces baroreceptor sensitivity in healthy humans and dogs. The mechanisms by which neurohormonal activation reduces baroreceptor sensitivity remain unclear [54,55]. This can lead to an elevated heart rate, which over time contributes to the worsening of heart failure [56,57]. Elevated heart rate is an independent predictor of poor prognosis in patients with heart failure [54,55,58].

#### Biomarkers and Potential Target Therapy

Inflammatory markers and anti-inflammatory therapies have been extensively reviewed throughout the past decade [59,60,61]. A comprehensive analysis of these topics falls outside the scope of this review. In this section, we summarize the biomarkers of inflammation and their relation to HF.

**Figure 3:**
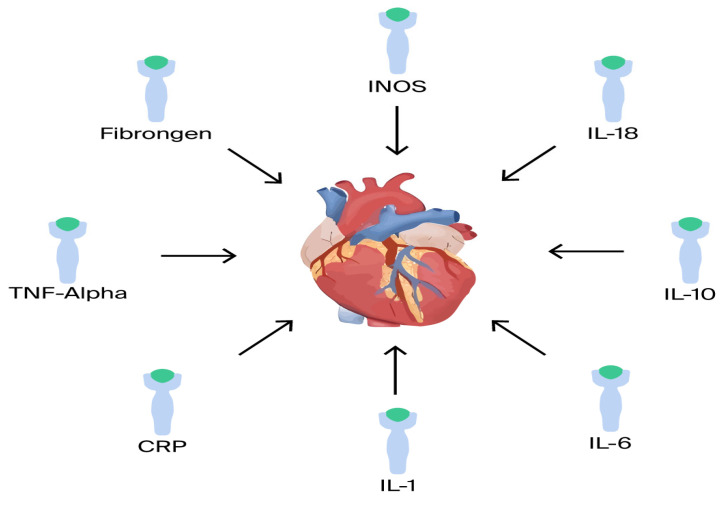
Proinflammatory mediators.

Inflammatory cytokines: There is a correlation between increased severity of HF and pro-inflammatory cytokines [62]. Several pro- and anti-inflammatory cytokines have been shown to play a major role in HF, including TNF-α, IL-1, IL-6, IL-8, IL-18, IL-1RA, and IL-33 (Figure 3). For example, in the BIOSTAT-CHF trial, analysis of a comprehensive multi-marker profiling panel revealed that biological processes producing or responding to interferon-gamma (IFN-γ) correlate with reduced mortality, whereas processes associated with T-cell activity demonstrate a relationship with increased mortality rates [20].

TNF-α: TNF-α is one of the most studied cytokines in HF. It is produced by many cell types, such as macrophages, cardiomyocytes, and endothelial cells [59]. Elevated levels of TNF-α have been found in patients with HFrEF, HFpEF, acute HF, and cardiogenic shock [63]. TNF-α mediates several heart-related effects, such as negative inotropic effect on cardiomyocytes by reducing cytosolic calcium levels [60]; inducing cell apoptosis [60]; cardiomyocyte hypertrophy [61]; and decreasing contractility [61]. Moreover, in a vicious cycle, TNF-α further triggers the sympathetic nervous system to release catecholamines, which in turn raise the level of TNF-α [64,65,66].

The therapeutic strategy of targeting tumor necrosis factor-α (TNF-α) in chronic heart failure (CHF) has been evaluated in multiple clinical trials with discouraging outcomes. Both the RENEWAL and RECOVER trials, which investigated etanercept, were prematurely terminated due to safety concerns [66]. Similarly, disappointing results were observed in the ATTACH trial examining infliximab [67], further highlighting the ineffectiveness of this approach in CHF management.

IL-1: A primary cytokine involved in the initiation of HF inflammation [68].

Elevated levels of IL-1 have been shown in patients with CHF, regardless of the etiology. This cytokine, produced by cardiomyocytes, immune cells, endothelial cells, and fibroblasts, has various effects on cardiac tissue. These include the induction of cardiomyocyte apoptosis, increased fibrosis, and cardiac remodeling [59]. IL-1 also disrupts mitochondrial energy production, causing a dysfunction of myocardial inotropism [69].

Several trials evaluated the effectiveness of anti-IL-1 therapy. Most of these trials (D-HART, RED-HART, AIR-HF) demonstrated reduced inflammatory markers (e.g., CRP), but without influence on MACE or HF hospitalization [70,71,72]. The CANTOS trial (canakinumab, an interleukin-1β inhibitor) showed a lower rate of cardiovascular events [73]. Other clinical trials targeting various cytokines, including IL-6, IL-8, IL-18, IL-1RA, and IL-33, also showed disappointing results without significant clinical effect on MACE, HF hospitalizations, or other cardiovascular outcomes [74].

C-reactive protein: In addition to serving as a significant cardiovascular risk factor, C-reactive protein (CRP), particularly high-sensitivity CRP (hs-CRP), functions as an important prognostic indicator for cardiovascular risk stratification. Elevated CRP levels are associated with increased risk for major cardiac events and mortality, making it a valuable biomarker in clinical assessment [75]. Lakhani et al. reviewed the role of CRP in HFpEF and highlighted that CRP could be used as a biomarker to predict progression of HFpEF and other long-term clinical outcomes [76].

The prognostic values of CRP and statins in HFrEF and HFpEF patients were evaluated in several trials. TIME-CHF showed higher inflammation activity in heart failure patients regardless of ejection fraction (EF). Patients with stable chronic HF exhibited an average CRP level of 6.6 mg/L. The heightened systemic inflammatory response was even more pronounced in those with acute HF, as shown in the ASCEND-HF trial [77]. CRP is strongly and independently associated with HF in males. Conversely, for females, the association between CRP and HF is weaker [78]. Furthermore, statins may be beneficial in cases of HF with elevated CRP [79]. A post-hoc, not prespecified analysis of the CORONA trial suggested that statin therapy may reduce CRP levels and decrease hospitalization rates among patients with heart failure [80]. The COLCOT (Colchicine Cardiovascular Outcomes Trial) [81] examined the effectiveness of low-dose colchicine in patients with recent MI. The study demonstrated that colchicine significantly reduced the risk of ischemic cardiovascular events (including stroke and angina) and reduced CRP levels; however, it should be noted that the trial did not specifically investigate heart failure outcomes. The COLICA (Colchicina en Insuficiencia Cardiaca Aguda) trial focused specifically on acute heart failure and found that while colchicine effectively reduced inflammatory markers (CRP, IL-6), it failed to demonstrate significant improvements in heart failure clinical outcomes [82].

iNOS (Inducible Nitric Oxide Synthase): Nitric oxide (NO) is a diffusible free radical gas, inducible in two types of isoforms: iNOS or NOS2, and is present in nerve endings [4]. The impact of nitric oxide (NO) in the heart includes an inhibition of the positive inotropic effect of beta-adrenergic stimulation in cases of left ventricular (LV) dysfunction [83]. Evidence suggests that inducible nitric oxide synthase (iNOS) is associated with significant oxidative stress and insulin resistance, both of which are known to contribute to the pathophysiology of HFpEF [84].

Additionally, several studies indicate substantial iNOS activity in the context of both dilated cardiomyopathy (DCM) and ischemic cardiomyopathy [83,85]. Several trials, such as TRIUMPH, SHOCK-2, and LINCS [86,87,88] did not demonstrate that iNOS inhibitors significantly impact mortality or HF hospitalization rates.

Other factors, such as myeloperoxidase (MPO) [89], fibrinogen [90], and other inflammatory markers, have been associated with high inflammatory activity in patients with HF. However, most related targeted therapy trials yielded disappointing results, with no proven effect on mortality or clinical improvement.

The IL-33/ST2 axis: Interleukin-33 (IL-33), part of the interleukin-1 cytokine family, primarily plays a role in triggering a type 2 helper T cell (Th2) immune response by interacting with its receptor ST2 [91]. The IL-33/ST2 signaling pathway is mainly linked to autoimmune and inflammatory disorders, yet it also plays a central role in cardiovascular function and pathology. ST2 becomes elevated in rat cardiomyocytes under mechanical strain [92], while IL-33, its active ligand, is secreted by stretched cardiac fibroblasts and provides a cardioprotective role during myocyte strain-induced damage [93]. In individuals with advanced chronic heart failure, serum soluble ST2 (sST2) concentrations are markedly elevated and show a positive correlation with B-type natriuretic peptide (BNP) levels. Moreover, temporal fluctuations in sST2 levels serve as an independent prognostic indicator for future risk of death or the necessity of cardiac transplantation [94].

In the PARADIGM-HF trial, researchers explored how heart failure medications affect the levels of the biomarker sST2, which is linked to cardiac stress and inflammation. The study compared patients treated with sacubitril/valsartan versus those receiving enalapril, assessing sST2 at three intervals: at the start, one month, and eight months after randomization. Additionally, the trial analyzed how initial sST2 levels correlated with major clinical outcomes—namely, heart failure hospitalizations, cardiovascular deaths, and the combination of both. Patients taking sacubitril/valsartan generally experienced larger declines and fewer rises in sST2 compared to enalapril.

ST2 increase at 1 month was associated with poorer long-term prognosis, while sST2 decrease was associated with better outcomes [95].

## 3. Discussion

In this review, we elaborated and summarized the pathophysiological connection between inflammation and heart failure, illustrating the different types and mechanisms of inflammation. Despite numerous studies, the exact mechanisms linking heart failure and inflammation remain elusive, and further research is needed.

On the other side of the equation, many trials tried to demonstrate the impact of anti-inflammatory therapy on heart failure. Both the COLCOT and CANTOS trials have shown promising results in demonstrating the potential benefits of anti-inflammatory treatments in the context of cardiovascular disease. The results, specifically focusing on heart failure, have been less conclusive. Heart failure, with its various phenotypes and underlying mechanisms, makes it challenging to achieve encouraging outcomes; more targeted and customized approaches are warranted. The anti-TNF-α treatment has demonstrated high efficacy in inflammatory diseases such as Crohn’s and ankylosing spondylitis, yet understanding the failures and therapeutic insights from treatment protocols regarding it may help to create new directions in heart failure therapy. For example, in the ATTACH trial, they did not monitor the titers of the anti-drug antibodies. Advancing in 28 weeks, maybe monitoring these titers will help to create a better clinical outcome.

Secondly, many trials have focused on patients with moderate to severe heart failure, potentially missing a crucial window for intervention. Recognizing that inflammation in heart failure is a long-term process that can cause partially irreversible damage in later stages, future studies might benefit from selecting patients with new-onset or mild-to-moderate heart failure.

## 4. Conclusions

Inflammation plays a certain role in the pathogenesis and progression of heart failure. It is a multifaceted process involving the activation of the immune system, both innate and adaptive. Although reducing inflammation proves beneficial in ischemic heart disease, it does not yield the same therapeutic effect in heart failure of non-ischemic origin. Future studies are needed to further explore whether more selective anti-inflammatory therapies may improve outcomes in this large patient population.

## Figures and Tables

**Table 1:** Classification of heart failure. HF—heart failure, LVEF—left ventricular ejection fraction, HFrEF—heart failure with a reduced ejection fraction, HFmrEF—heart failure with a mildly reduced ejection fraction, HFpEF—heart failure with a preserved ejection fraction. ** Risk factors for people in this stage include hypertension, coronary vascular disease, diabetes, obesity, exposure to cardiotoxic agents, genetic variants for cardiomyopathy, and a family history of cardiomyopathy.

LVEF		Staging		NYHA	
HFrEF	LVEF ≤ 40	A	At risk ** for HF with no symptoms (currently or in the past) or structural or functional heart disease	I	Normal Activity
HFmrEF	LVEF 41–49	B	Patients with structural heart disease, evidence of increased cardiac filling pressures with no HF symptoms, and no symptoms in the past	II	HF symptoms at ordinary physical activity
HFpEF	LVEF ≥ 50	C	Symptomatic HF—People with current or previous symptoms of HF	III	HF symptoms at less than ordinary activity
		D	Advanced HF—Refractory HF symptoms that interfere with daily life functions or lead to repeated hospitalizations	IV	HF symptoms at rest

**Table 2:** Inflammatory disease and atherosclerosis.

Traditional CV Risk Factors	Hyperlipidemia, Hypertension, Obesity, Smoking, Diabetes
Chronic and acute mental stress	Autonomic nervous system
Acute infections	Urinary tract infection, endotoxin (gut microbiota)
Chronic infections	Bronchitis, periodontitis
Chronic autoimmune disease	Rheumatoid arthritis, systemic lupus, psoriasis, inflammatory bowel syndrome
Viral infections	COVID-19, influenza
Aging	Bone marrow activation and clonal hematopoiesis
Tissue injury	Myocardial infarction, chronic skin ulcers

## Data Availability

The original data presented in the study are openly available in https://pubmed.ncbi.nlm.nih.gov/; https://www.nejm.org/; https://www.jacc.org/; https://www.thelancet.com/.
